# Pursuing dynamics of minimal residual leukemic subclones in relapsed and refractory acute myeloid leukemia during conventional therapy

**DOI:** 10.1002/cam4.7182

**Published:** 2024-04-09

**Authors:** Dongchan Kim, Sheehyun Kim, Hyojin Song, Daehyeon Gwak, Suji Min, Ja Min Byun, Youngil Koh, Junshik Hong, Sung‐Soo Yoon, Hongseok Yun, Dong‐Yeop Shin

**Affiliations:** ^1^ Cancer Research Institute Seoul National University College of Medicine Seoul Republic of Korea; ^2^ Center for Medical Innovation Seoul National University Hospital Seoul Republic of Korea; ^3^ Center for Precision Medicine Seoul National University Hospital Seoul Republic of Korea; ^4^ Department of Internal Medicine Seoul National University Hospital Seoul Republic of Korea

**Keywords:** acute myeloid leukemia, cancer biology, leukemia, molecular cytogenetics

## Abstract

**Background:**

Acute myeloid leukemia (AML) is characterized by clonal heterogeneity, leading to frequent relapses and drug resistance despite intensive clinical therapy. Although AML's clonal architecture has been addressed in many studies, practical monitoring of dynamic changes in those subclones during relapse and treatment is still understudied.

**Method:**

Fifteen longitudinal bone marrow (BM) samples were collected from three relapsed and refractory (R/R) AML patients. Using droplet digital polymerase chain reaction (ddPCR), the frequencies of patient's leukemic variants were assessed in seven cell populations that were isolated from each BM sample based on cellular phenotypes. By quantifying mutant clones at the diagnosis, remission, and relapse stages, the distribution of AML subclones was sequentially monitored.

**Results:**

Minimal residual (MR) leukemic subclones exhibit heterogeneous distribution among BM cell populations, including mature leukocyte populations. During AML progression, these subclones undergo active phenotypic transitions and repopulate into distinct cell population regardless of normal hematopoiesis hierarchic order. Of these, MR subclones in progenitor populations of patient BM predominantly carry MR leukemic properties, leading to more robust expansion and stubborn persistence than those in mature populations. Moreover, a minor subset of MR leukemic subclones could be sustained at an extremely low frequency without clonal expansion during relapse.

**Conclusions:**

In this study, we observed treatment persistent MR leukemic subclones and their phenotypic changes during the treatment process of R/R AML patients. This underscores the importance of preemptive inhibition of clonal promiscuity in R/R AML, proposing a practical method for monitoring AML MR subclones.

## 1 INTRODUCTION

Acute myeloid leukemia (AML) is a hematologic malignancy characterized by heterogeneous molecular and cytogenetic profiles.^1^, ^2^ AML clonal diversity, which is strongly associated with cytogenetic abnormalities, is known to cause difficulties in achieving AML cure.^3^, ^4^, ^5^ Although evidence such as clinical, molecular/genetic, morphologic, and immunophenotypic parameters has accumulated for a long time,^6^, ^7^, ^8^ elucidating their clinical relevance in terms of AML recurrence and treatment persistence at the subclonal level remains a challenge.

The current treatment for AML patients at an early stage has been standardized as high‐intensity, cytotoxic drug combination therapy for the past 40 years.^6^, ^9^ Initial treatment can induce temporary remission in approximately 60%–80% of younger and 40%–60% of older AML patients,^10^ but relapse occurs in most patients. Due to this unmet medical need, recent AML studies have addressed specific subclones accompanied by heterogeneous molecular cytogenetics, leading AML to relapse and treatment resistance.^11^, ^12^, ^13^ Thus, understanding the heterogeneous clonal architecture of AML that evolves during hematopoiesis is indispensable for identifying profitable treatment strategies for AML.

In relapsed and refractory (R/R) AML patients, pre‐existing leukemic subclones, such as leukemic stem cells (LSCs), are known to drive AML recurrence. LSCs reside in the normal stem progenitor populations of patient BM, and their stem‐like properties, such as self‐renewal in the dormant state, give rise to drug‐resistant mutant subclones after treatment.^14^, ^15^, ^16^, ^17^ Various efforts using high‐resolution multiomics approaches to identify AML minimal residual (MR) drug‐resistant clones, including LSCs, have been actively conducted,^18^, ^19^, ^20^, ^21^ practical attempts to pursue this identification in patients during clinical procedures have not been fully investigated.

Here, we chronologically investigate drug‐resistant MR leukemic subclones in AML patients throughout the treatment process. The practical approach employed in this study, utilizing droplet digital polymerase chain reaction (ddPCR) assay combined with multiparameter immunophenotyping, enables us to explore the clonal promiscuity among MR leukemic cells in the bone marrow of R/R AML patients.

## 2 MATERIALS AND METHODS

### 2.1 Clinical samples

Bone marrow (BM) aspirates, matched saliva samples of relapsed and therapy‐refractory AML patients, and donor‐mobilized peripheral blood (mPB) were obtained with informed consent under the approval of the Seoul National University Hospital (SNUH) Institutional Review Board (IRB No. 1201‐099‐396). Mononucleated cells (MNCs) of patient samples were isolated by Ficoll gradient (17‐1440‐02, Ficoll® Paque Plus, Cytiva) centrifugation and archived in institutional repositories. Cryopreserved MNCs were thawed in a 37°C water bath, washed with Dulbecco's phosphate‐buffered saline (LM 001‐02, DPBS, Welgene), and used for analysis. Genomic DNA was extracted from MNCs using QIAamp DNA Blood Kits (51104, QIAGEN, Hilden, Germany) and from matched saliva using a saliva DNA collection kit (RU49000, Norgen Biotek, Ontario, Canada) and saliva gDNA isolation kit (RU45400, Norgen Biotek, Ontario, Canada), according to the manufacturer's instructions.

### 2.2 Whole exome sequencing

Whole exome sequencing (WES) was performed on BM aspirates and matched saliva samples from R/R AML patients. Genomic DNA was extracted from the BM aspirates and matched saliva samples, and DNA libraries were constructed using the SureSelect XT Human All Exon Kit (V5) (Agilent Technologies, Santa Clara, CA). Paired‐end sequencing was performed using the NovaSeq 6000 System (Illumina Inc., San Diego, CA, USA).

### 2.3 Somatic variant screening

Sequenced reads were aligned to the reference human genome (GRCh37/hg19) using the Burrows–Wheeler Aligner (BWA v0.7.17) and GATK Best Practice (v4.0.2.1). Single‐nucleotide variants (SNVs) and small insertions and deletions (indels) were detected using an in‐house developed pipeline, SNVer (v0.5.3), and LoFreq (v2.1.2). Mutations were annotated using SnpEff (v5.0e) and cross‐checked using Vardict (v1.8.2) and GATK4 Mutect2. The variant filter conditions for true variant selection were >5 altered evidence reads and VAF >5%. Variants with population frequencies over 0.1% in the Genome Aggregation Consortium (gnomAD) East Asian database were also filtered out. Eleven leukemic or likely leukemic somatic variants of three patients were finally screened through WES‐variant screening.

### 2.4 Fluorescence‐activated cell sorting of hematopoietic cell fractions

Archived BM MNCs were thawed quickly and stained with the following fluorescent‐conjugated antibodies: anti‐human CD45‐FITC (560976, BD Pharmingen), CD34‐APC (560940, BD Pharmingen), CD38‐BV421 (562445, BD Horizon), CD45RA‐BV711 (563733, BD Horizon), CD90‐PE (555596, BD Pharmingen), and CD123‐PE‐Cy7 (560826, BD Pharmingen); and three leukocyte populations: CD45‐FITC, CD33‐PE (561816, BD Pharmingen), CD3‐PE‐Cy7 (560910, BD Pharmingen), and CD19‐PerCP‐Cy5.5 (340951, BD Bioscience). Using a BD AIRAIII cell sorter (BD Bioscience), MNCs were sorted into seven hematopoietic cell fractions: four hematopoietic stem progenitors (HSPCs) of CD45+ cell fractions: HSCs/MPPs (hematopoietic stem cells/multipotent progenitors, CD34 + CD38‐CD90+/‐CD45RA‐), LMPPs (lymphoid‐primed multipotent progenitors, CD34 + CD38‐CD90‐CD45RA+), CMPs/MEPs (common myeloid progenitors/megakaryocyte erythroid progenitors, CD34 + CD38 + CD45RA‐CD123+/−), and GMPs (granulocyte monocyte progenitors, CD34 + CD38 + CD45RA + CD123+), and three mature leukocytes: CD45dim myeloid cells (CD33 + CD3‐), CD45 bright T cells (CD3 + CD19‐), and B cells (CD3‐CD19‐). (Figure S1).

### 2.5 Droplet digital PCR

For single nucleotide polymorphism (SNP) detection, genomic DNA was extracted from 7 sorted cell fractions of patient samples using a REPLI‐g Mini Kit (150,023, QIAGEN, Hilden, Germany). Following the manufacturer's instructions, the concentrations of sorted cells were adjusted to 600 cells/μL in 0.5 μL and reacted overnight for whole genome amplification. To investigate patient specific leukemic clones within the AML BM pool that may contribute to relapse and treatment resistance in patients, we selected six representative leukemic variants demonstrating significant changes during the patient treatment process among the 11 leukemic variants. Total six variant‐specific TaqMan probe assays were designed for the ddPCR assay (Table S2). Variant‐specific TaqMan probe assays for two representative leukemic variants from each patient were designed for the ddPCR assay (Table S2). Before analyzing samples, assay conditions were optimized by performing serially diluted wild‐type DNA loading, and “no template controls” (NTCs) were run in parallel to avoid amplicon contamination issues. Wild‐type and altered sequences were detected in the amplified gDNA using a fluorescein/hexachloro (FAM/HEX) two‐color detection ddPCR system (QX200, Bio‐Rad). Variant allele frequency (VAF) values for each target were calculated as the percentage (%) of positive droplets divided by the sum of positive and negative droplets (total accepted droplets), indicating the frequency of variant clones. We noted the minimum detection level of the ddPCR assay (at least three dots, VAF >0.1%).^14^ However, because we sought to observe the eradication and repopulation of MR subclones, all data are presented in our results.

## 3 RESULTS

### 3.1 Detection of leukemic subclones in longitudinally collected patient samples

We first collected three relapsed and refractory (R/R) AML patients (*n* = 3) who initially received conventional chemotherapy. As conventional chemotherapy, standard 7 + 3 induction was administered to these three patients, and complete remission (CR) was achieved. They received 3 cycles of high‐dose ara‐C consolidation or allogeneic peripheral blood hematopoietic stem cell transplantation (allo‐PBSCT), but relapsed within a year. After reinduction chemotherapy, second remission (CR2) was achieved in three patients. They received additional donor lymphocyte infusion (Patient #1) or allo‐PBSCT (Patients #2 and #3) as consolidation therapy, but Patients #1 and #2 showed treatment persistency (Per) after secondary relapse (Rel2), and Patient #3 showed mixed chimerism suspicious of impending relapse (Table 1).

**TABLE 1:** Clinical characteristics and outcomes of three patients with relapsed and refractory AML.

Patient's No.	Sex/age	Diagnosis	Induction regimen	Therapy after CR1	PFS1	Re‐induction regimen	Therapy after CR2	PFS2	OS	Cause of death
1	F/45	AML, M4	7 + 3	MSD‐allo‐PBSCT	5.5 months	FLAG‐I	Donor lymphocyte infusion	20 months	30 months	Sepsis
2	M/55	AML with RUNX1‐RUNX1T1	7 + 3	3 cycles of HDAC	9.5 months	FLAG‐I	Haploidentical PBSCT	2.5 months	19 months	Sepsis with leukemia
3	M/39	AML with RUNX1‐RUNX1T1	7 + 3	3 cycles of HDAC	11 months	CLAG‐M	MUD‐allo‐PBSCT	20 months	37 months	GVHD and sepsis

Abbreviations: 7 + 3, 7 days of cytarabine arabinoside (ara‐C) and 3 days of idarubicin; Allo‐PBSCT, allogeneic peripheral blood hematopoietic stem cell transplantation; AML, acute myeloid leukemia; CLAG‐M, combination chemotherapy of cladribine, ara‐c, G‐CSF, and mitoxantrone; F, female; FLAG‐I, combination chemotherapy of fludarabine, ara‐c, granulocyte colony stimulating factor (G‐CSF), and idarubicine; GVHD, graft‐versus‐host disease; HDAC, high dose of ara‐C consolidation; M, male; MSD, matched sibling donor; MUD, matched unrelated donor; OS, overall survival since the initial diagnosis of AML; PFS1, progression‐free survival (PFS) since the achievement of complete remission (CR) after the 1st induction treatment to 1st relapse; PFS2, PFS since the achievement of the secondnd CR after re‐induction treatment to second relapse.

From 3 R/R AML patients, 15 longitudinally collected BM total nucleated cell (TNC) samples were organized (Table S1), and 8 out of 15 organized samples were used for somatic variant screening. Among the 11 screened oncogenic (or likely oncogenic) variants, 6 out of 11 leukemic variants (Patient #1: *FLT3* ^*D835E*(*A*>*C*)^, *WT1* ^*R375P*^; Patient #2: *KIT* ^*D816Y*^, *ASXL1* ^*A722L*^; Patient #3: *KIT* ^*N822K*^, *FLT3* ^*D835E*(*A*>*T*)^) showing significant changes during the treatment process were finally selected (Table 2). The frequency of leukemic clones with selected leukemic variants in seven sorted BM cell fractions (four stem/progenitors: HSCs/MPPs, LMPPs, CMPs/MEPs, and GMPs; three mature leukocytes: myeloid cells, T lymphocytes, and B lymphocytes) (Figure S1) was determined using the designed probe assay. Based on the measured variant allele frequency (VAF) values, we examined the clonal distribution of leukemic subclones in the BM of patients with R/R AML.

**TABLE 2:** Allele frequency of oncogenic (or likely oncogenic) variants assessed by whole exome sequencing in three relapsed and refractory AML patients.

Patient	Cytogenetics	Variant	Chr	Position	Ref	Alt	Reference Gene	Dx	Rel1	Rel2	Per	OncoKB
1	M4, FLT3‐TKD	FLT3‐D835E	13	28592640	A	C	NM_004119	0.462	0.319	0.332	0.497	Oncogenic
		PHF6‐H136Qfs*2	X	1.34E+08	‐	A	NM_001015877	0.401	0.393	0.378	0.437	Likely oncogenic
		RUNX1‐A149Rfs*11	21	36252917	‐	G	NM_001754	0.492	0.396	0.385	0.425	Likely oncogenic
		WT1‐R375Pfs*6	11	32417943_32417945	CGT	GCGGC	NM_024426	0.000	0.295	0.405	0.440	Likely oncogenic
2	t (8;21) (q22;q22)	ASXL1‐A772Lfs*4	20	31022829	G	‐	NM_015338	0.000	0.317	‐	‐	Likely oncogenic
		FLT3‐D835V	13	28592641	T	A	NM_004119	0.100	0.000	‐	‐	Oncogenic
		KIT‐D816Y	4	55599320	G	T	NM_000222	0.000	0.365	‐	‐	Oncogenic
		NRAS‐Q61R	1	1.15E+08	T	C	NM_002524	0.057	0.000	‐	‐	Oncogenic
3	t (8;21) (q22;q22)	FLT3‐D835E	13	28592640	A	T	NM_004119	0.064	0.000	‐	‐	Oncogenic
		KIT‐N655K	4	55594262	T	G	NM_000222	0.054	0.000	‐	‐	Likely oncogenic
		KIT‐N822K	4	55599340	T	G	NM_000222	0.193	0.446	‐	‐	Oncogenic

*Note*: Patient #1 samples at Dx, CR1, Rel1, CR2, Rel2, and Per; Patient #2 at Dx, CR1, Rel1, and CR2; Patient #3 at Dx, CR1, Rel1, CR2, and CR2 (post‐PBSCT).

Abbreviations: Alt, alternate, Chr, chromosome; CR, complete remission; Dx, diagnosis; Per, persistance; Ref, reference; Rel, relapse.

### 3.2 Treatment‐persistent leukemic subclones exhibit versatile phenotypes during treatment

**FIGURE 1:**
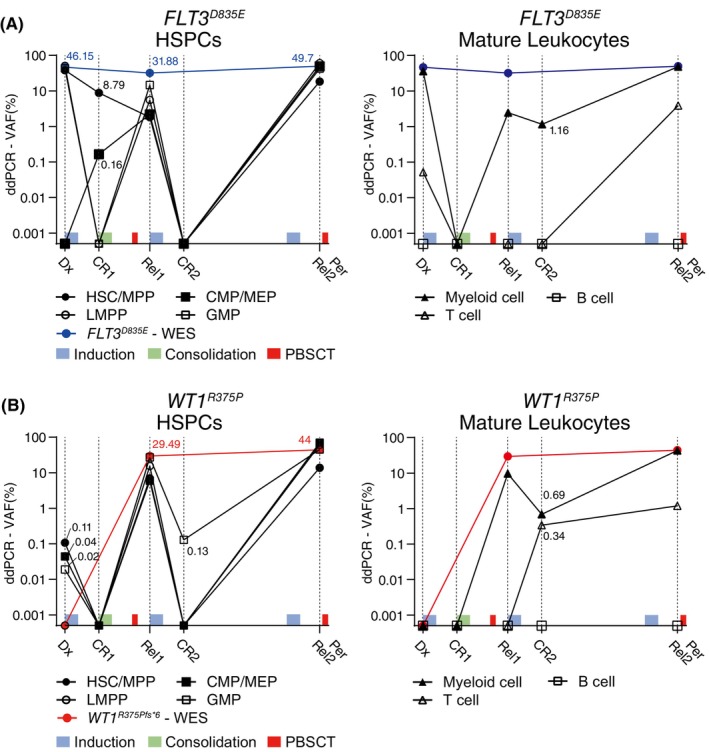
The distribution of *FLT3* ^*D835E*^ and *WT1* ^*R375P*^ mutant clones from 7 bone marrow (BM) cell fractions in Patient #1. Cells from longitudinal AML BM samples were sorted by phenotype into 7 cell fractions (4 HSCP populations and 3 mature leukocytes) using flow cytometry. The frequencies of *FLT3* ^*D835E*^ and *WT1* ^*R375P*^ mutant clones in the sorted cell fractions were determined using ddPCR. Light blue: induction therapy; green: consolidation therapy; red: allogeneic G‐CSF‐mobilized peripheral blood stem cell transplantation. (A) Frequency of the *FLT3* ^*D835E*^ mutant clone (variant allele frequency, VAF) distributed in HSCs/MPPs, LMPPs, CMPs/MEPs, GMPs (left), myeloid cells, T cells, and B cells (right) during treatment. The blue line represents the VAF in the entire BM obtained by the bulk WES. (B) Frequency of the *WT1* ^*R375P*^ mutant clone distributed in seven cell fractions during treatment. The red line represents the fraction of mutant clones in the total bone marrow identified by bulk WES.

Patient #1 exhibited a varied distribution of *FLT3* ^*D835E*(*A>C*)^ mutant leukemic clones across different BM cell populations (HSCs/MPPs, LMPPs, GMPs, myeloid cells, and T cells; VAF:38.1%, 50.8%, 42.9%, 36.1%, and 0.05%, respectively) at diagnosis (Dx). Notably, after the initial treatment, mutant clones in the HSC/MPP population displayed treatment persistence (8.79%), while those in LMPPs, GMPs, myeloid cells, and T cells were effectively reduced. Moreover, these treatment‐persistent clones were newly repopulated into distinct cell populations (CMPs/MEPs: 0.165%), in which no mutant clones were initially detected. After clonal expansion of Rel1, these persisting clones were effectively reduced through relapse treatment, yet they remained only in the mature leukocyte population (myeloid cells: 1.16%) (Figure 1A; Figure S2A).

In the ddPCR analysis of Patient #1, the clones with leukemic variant *WT1* ^*R375P*^, which were not detected by NGS analysis, were initially found at very low frequencies in the HSC/MPP, CMP/MEP, and GMP (0.11, 0.04, and 0.02%, respectively) populations. Despite the initial treatment leading to an overall reduction in mutant clones at CR1, these mutant clones unexpectedly resurfaced and expanded across all seven BM cell populations at Rel1. The *WT1* ^*R375P*^ mutant clones in GMP and myeloid cell populations demonstrated treatment persistence after relapse treatment, similar to *FLT3* ^*D835E*^ mutant clones. Moreover, these persisting clones repopulated another mature leukocyte population (T cells: 0.34%), even after achieving a second remission (CR2) (Figure 1B; Figure S2A).

In Patient #1, *FLT3* ^*D835E*(*A*>*C*)^ and *WT1* ^*R375P*^ mutant in some cell populations display treatment persistence and dynamic changes in their cellular phenotypes throughout treatment. These leukemic subclones were likely indicative of minimal residual (MR) disease cells in Patient #1 with R/R AML.

### 3.3 Leukemic subclones with primitive phenotypes are the main carriers of MR disease properties

**FIGURE 2:**
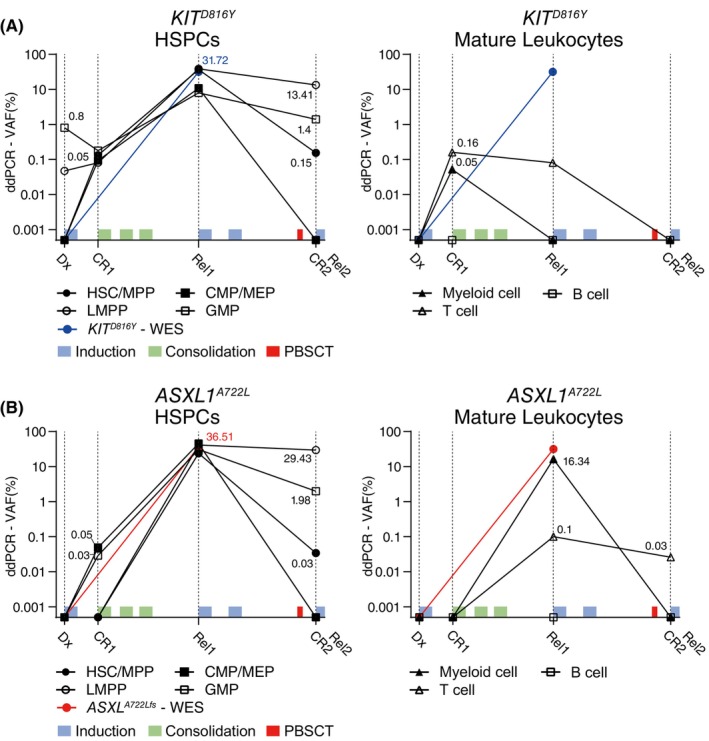
*KIT* ^*D816Y*^ and *ASXL1* ^*A722L*^ mutant clones distributed in 7 BM cell fractions of Patient #2. AML BM cells were analyzed and sorted into 7 cell fractions according to their phenotype profiles. Allele frequencies of *KIT* ^*D816Y*^ and *ASXL1* ^*A722L*^ variants, indicating the fractions of mutant clones, were determined using a ddPCR assay. Light blue: induction therapy; green: consolidation therapy; red: allogeneic G‐CSF‐mobilized peripheral blood stem cell transplantation. (A) Changes in the frequency of the *KIT* ^*D816Y*^ mutant clone determined by ddPCR in 7 cell fractions during treatment. The blue line represents the VAF in the entire BM obtained by the bulk WES. (B) Changes in the frequency of the *ASXL1* ^*A722L*^ mutant clone in 7 cell fractions were determined by ddPCR. The red line represents the fraction of mutant clones in the total bone marrow identified by bulk WES.

In Patient #2, in contrast to the NGS analysis, ddPCR analysis showed that the clones with the *KIT* ^*D816Y*^ leukemic variant resided only in the LMPP and GMP (0.05% and 0.8%, respectively) populations at an extremely low frequency. As observed in Patient #1, these few mutant clones exhibited persistence following initial treatment and repopulated actively across all progenitors as well as mature leukocytes at CR1. Among these, treatment‐persistent clones in progenitor populations tended to show active clonal expansion at Rel1, whereas those in mature leukocytes showed a significant reduction. These persister clones eventually remained only in the HSCs/MPPs, LMPPs, and GMPs (0.15%, 13.42%, and 1.39%, respectively) after relapse treatment at CR2 (Figure 2A; Figure S2B).

The *ASXL1* ^*A722L*^ mutant leukemic clone of Patient #2 was absent at initial diagnosis (Dx). However, the mutant clones appeared in CMPs/MEPs and GMPs at CR1 and subsequently in all BM cell populations, except for the B cell population, regardless of the treatment. Similar to the above *KIT* ^*D816Y*^ mutant clones, the *ASXL1* ^*A722L*^ mutant clones showing treatment persistence with repopulating capacity exhibited varied responses to relapse treatment. Although relapse treatment and allo‐PBSCT effectively suppressed mutant clones in mature leukocytes (myeloid cells), mutant clones in the progenitor populations persisted and eventually remained in HSCs/MPPs, LMPPs, and GMPs (0.03%, 29.48%, 1.98%, and 0.03%, respectively) (Figure 2B; Figure S2B).

In Patient #2, the persister clones occupying the progenitor populations seemed to show relatively robust clonal expansion and treatment persistence compared to those in mature leukocyte populations. The primitive BM cell populations are likely to carry treatment‐persistent MR leukemic subclones securely in R/R AML patients.

### 3.4 Leukemic subclones can sustain MR disease properties without clonal expansion

**FIGURE 3:**
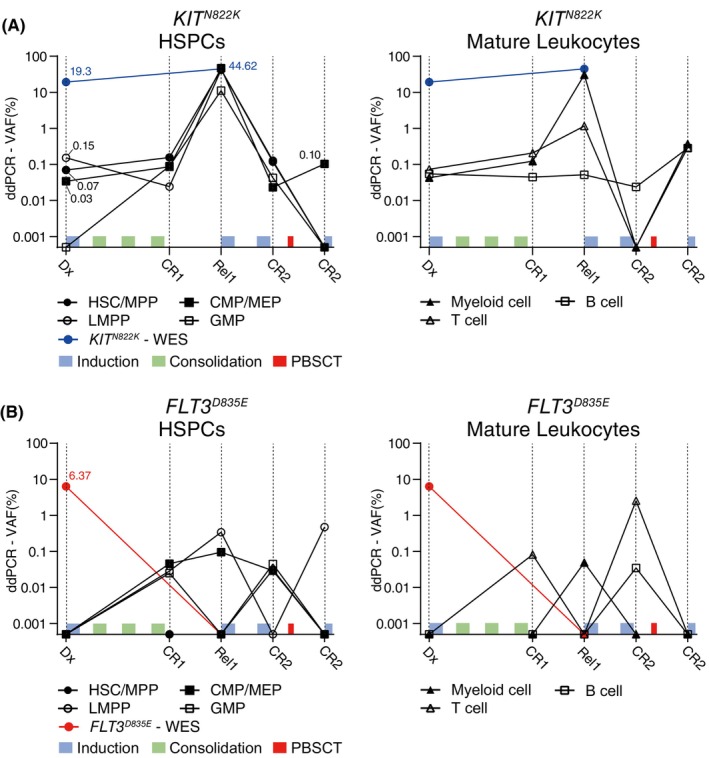
Distribution of *KIT* ^*N822K*^ and *FLT3* ^*D835E*^ mutant clones and BM constitution in Patient #3. The BM cells were sorted into 7 cell fractions based on their cellular phenotypes. Variant allele frequencies of mutant cells were determined in genomic DNA obtained from 7 sorted cell fractions using ddPCR. Light blue: induction therapy; green: consolidation therapy; red: allogeneic G‐CSF‐mobilized peripheral blood stem cell transplantation. (A) Frequency changes in the *KIT* ^*N822K*^ mutant clone in 7 cell fractions were determined by ddPCR during treatment. The blue line represents the VAF in the entire BM obtained by the bulk WES. (B) Frequency changes in the *FLT3* ^*D835E*^ mutant clone in 7 cell fractions were determined by ddPCR. The red line represents the fraction of mutant clones in the total bone marrow identified by bulk WES.

In the ddPCR analysis of Patient #3, the *KIT* ^*N822K*^ mutant was observed at low frequencies in almost all BM cell populations. These mutant clones generally persisted after the initial treatment and subsequently repopulated even into a new population (GMPs: 0.09%) at CR1. These treatment‐persistent leukemic clones at Rel1 exhibited robust expansion in four progenitor and myeloid cell populations, with slight increases and persistence in T cells (0.2% to 1.15%) and B cells (0.04% to 0.05%). Although relapse treatment and allo‐PBSCT led to an overall reduction in the number of persistent clones, these clones eventually remained at low frequencies (CR2) (CMPs/MEPs, myeloid, T, and B cells: 0.10, 0.37, 0.29, and 0.28%, respectively) (Figure 3A; Figure S2C).

The *FLT3* ^*D835E(A>T)*^ mutant clone of Patient #3, detected at 6.37% in NGS analysis, was not observed in the ddPCR analysis of seven BM cell fractions at diagnosis (Dx). However, at first remission (CR1), mutant clones appeared in LMPPs, CMPs/MEPs, GMPs, and T cells (0.03, 0.05, 0.03, and 0.08%) at extremely low frequencies. In contrast to the other persisting clones, these treatment‐persistent clones, which showed no markable expansion (VAFs below 0.5%) at Rel1, were irregularly distributed across the seven populations at extremely low frequencies throughout the treatment process. (Figure 3B; Figure S2C).

Two treatment‐persistent clones in Patient #3 showing distinct changes in their VAF values may indicate the coexistence of two independent MR leukemic subclones. In addition, they can sustain MR disease properties throughout the treatment process by residing in various cell types at a low frequency without clonal expansion.

## 4 DISCUSSION

This study continuously monitored minimal residual (MR) disease clones in the bone marrow (BM) of patients with relapsed and refractory (R/R) AML, rather than identifying novel therapeutic targets of LSCs. Using NGS and ddPCR genotyping methods combined with multiparameter immunophenotyping analysis used in previous studies,^14^, ^19^, ^22^ the dynamics of AML MRD clones were traced throughout the patient's treatment process.

Using this combined approach, we sought to identify clones bearing leukemic variants within the BM cell population of patients. In three patients, some leukemic clones within the leukemic cell pool exhibited persistent responses to AML treatment. In addition, these treatment‐persistent clones were irregularly distributed in various types of cells, including mature leukocytes, and were greatly expanded in the relapse stages. We were able to identify these treatment‐persistent clones as MR leukemic subclones that might be associated with treatment refractoriness and recurrence of AML. However, owing to phenotypic heterogeneity, we were unable to categorize the MR leukemic subclones according to their cellular phenotypes in our three cases. This study showed chronological features of MR leukemic subclones during the treatment process of R/R AML in practical way.

To date, multiple studies have revealed that CD34+/CD38− cells represent the phenotype of pre‐existing drug‐resistant clones and leukemic stem cells (LSCs) in AML BM.^23^, ^24^ Consistent with this, our analysis showed that CD34‐expressing MR leukemic subclones of primitive phenotypes (HSCs/MPPs, LMPPs, CMPs/MEPs, and GMPs) actively repopulated other cell populations. MR leukemic subclones with primitive phenotypes appeared to be an initial contributor not only carrying MR disease properties themselves but also transmitting them to other populations with distinct phenotypes. Our results demonstrated that CD34‐expressing AML MR clones partially represented a small number of true LSCs that withstood AML treatment and expanded greatly in relapse/treatment persistent stages.^25^

In this study, we hypothesized that specific clones with leukemic variants showing significant changes after the clinical remission stage might contribute to AML relapse and acquire treatment resistance as MRD‐like clones in the BM. Therefore, we investigated two representative leukemic clones in each patient and demonstrated two features of MR subclones: (1) a small number of preexisting MR leukemic subclones and (2) dynamic changes in their phenotypes, regardless of the hierarchical order of normal hematopoiesis. Considering the clonal promiscuity of MR clones that emerge during these treatments, the study of a direct correlation between MR clones and AML relapse and drug resistance is left to our future studies.

By proposing a practical method to track MR leukemic subclones, this study reveals the clonal promiscuity of MR disease in the BM of R/R AML patients. We emphasize that proactive suppression of AML MRD clones is crucial for inhibiting AML onset.

## AUTHOR CONTRIBUTIONS

**Dongchan Kim:** Conceptualization (equal); data curation (lead); formal analysis (equal); investigation (lead); methodology (lead); validation (lead); visualization (lead); writing – original draft (lead); writing – review and editing (lead). **Sheehyun Kim:** Data curation (equal); formal analysis (supporting); investigation (lead); methodology (equal); software (equal); supervision (equal); validation (equal); visualization (equal); writing – original draft (equal). **Hyojin Song:** Data curation (lead); formal analysis (lead); investigation (equal); methodology (equal); software (lead); supervision (equal); validation (lead); visualization (supporting). **Daehyeon Gwak:** Formal analysis (supporting); investigation (supporting); methodology (supporting); validation (equal); writing – original draft (equal). **Suji Min:** Data curation (equal); formal analysis (equal); funding acquisition (equal); methodology (equal). **Ja Min Byun:** Funding acquisition (equal); project administration (equal); resources (equal); software (equal). **Youngil Koh:** Funding acquisition (equal); project administration (equal); resources (equal). **Junshik Hong:** Funding acquisition (equal); project administration (equal); resources (equal). **Sung‐Soo Yoon:** Funding acquisition (lead); project administration (equal); resources (lead); supervision (lead); writing – review and editing (equal). **Hongseok Yun:** Conceptualization (lead); data curation (lead); formal analysis (lead); funding acquisition (equal); investigation (lead); methodology (equal); project administration (equal); software (equal); supervision (lead); validation (equal); writing – original draft (equal); writing – review and editing (equal). **Dong‐Yeop Shin:** Conceptualization (lead); data curation (lead); formal analysis (equal); funding acquisition (lead); investigation (lead); methodology (equal); resources (lead); supervision (lead); validation (equal); writing – original draft (lead); writing – review and editing (lead).

## FUNDING INFORMATION

This work was supported by a National Research Foundation of Korea (NRF) grant funded by the Korean government (MIST) [grant number NRF‐2021R1F1A1048546] and by the Seoul National University Hospital [grant number 0420230560]

## CONFLICT OF INTEREST STATEMENT

The authors listed on this manuscript declare that they have no competing interests related to this study.

## Supporting information


Figure S1.

Figure S2.

Table S1.

## Data Availability

Data Availability StatementThe authors agree with the content of this manuscript. All data and additional information regarding the research procedure included in this study are available upon request from the corresponding author (Shindongyeop@snu.ac.kr). This study has not been submitted for publication elsewhere. .

## References

[1] Papaemmanuil E , Gerstung M , Bullinger L , et al. Genomic classification and prognosis in acute myeloid leukemia. N Engl J Med. 2016;374(23):2209‐2221. doi:10.1056/NEJMoa1516192 27276561 PMC4979995

[2] Welch JS , Ley TJ , Link DC , et al. The origin and evolution of mutations in acute myeloid leukemia. Cell. 2012;150(2):264‐278. doi:10.1016/j.cell.2012.06.023 22817890 PMC3407563

[3] McGranahan N , Swanton C . Clonal heterogeneity and tumor evolution: past, present, and the future. Cell. 2017;168(4):613‐628. doi:10.1016/j.cell.2017.01.018 28187284

[4] Cancer Genome Atlas Research N , Ley TJ , Miller C , et al. Genomic and epigenomic landscapes of adult de novo acute myeloid leukemia. N Engl J Med. 2013;368(22):2059‐2074. doi:10.1056/NEJMoa1301689 23634996 PMC3767041

[5] Min GJ , Cho BS , Park SS , et al. Treatment for relapsed acute promyelocytic leukemia: what is the best post‐remission treatment? Blood Res. 2022;57(3):197‐206. doi:10.5045/br.2022.2022060 35880495 PMC9492525

[6] Dohner H , Weisdorf DJ , Bloomfield CD . Acute myeloid leukemia. N Engl J Med. 2015;373(12):1136‐1152. doi:10.1056/NEJMra1406184 26376137

[7] Cheson BD , Cassileth PA , Head DR , et al. Report of the National Cancer Institute‐sponsored workshop on definitions of diagnosis and response in acute myeloid leukemia. J Clin Oncol. 1990;8(5):813‐819. doi:10.1200/JCO.1990.8.5.813 2185339

[8] Grimwade D , Hills RK . Independent prognostic factors for AML outcome. Hematology Am Soc Hematol Educ Program. 2009;2009:385‐395. doi:10.1182/asheducation-2009.1.385 20008224

[9] Othus M , Kantarjian H , Petersdorf S , et al. Declining rates of treatment‐related mortality in patients with newly diagnosed AML given ‘intense’ induction regimens: a report from SWOG and MD Anderson. Leukemia. 2014;28(2):289‐292. doi:10.1038/leu.2013.176 23760400 PMC4457325

[10] Rowe JM . Optimal induction and post‐remission therapy for AML in first remission. Hematology Am Soc Hematol Educ Program. 2009;2009:396‐405. doi:10.1182/asheducation-2009.1.396 20008225

[11] Parkin B , Ouillette P , Li Y , et al. Clonal evolution and devolution after chemotherapy in adult acute myelogenous leukemia. Blood. 2013;121(2):369‐377. doi:10.1182/blood-2012-04-427039 23175688 PMC3653567

[12] Ding L , Ley TJ , Larson DE , et al. Clonal evolution in relapsed acute myeloid leukaemia revealed by whole‐genome sequencing. Nature. 2012;481(7382):506‐510. doi:10.1038/nature10738 22237025 PMC3267864

[13] Eppert K , Takenaka K , Lechman ER , et al. Stem cell gene expression programs influence clinical outcome in human leukemia. Nat Med. 2011;17(9):1086‐1093. doi:10.1038/nm.2415 21873988

[14] Shlush LI , Mitchell A , Heisler L , et al. Tracing the origins of relapse in acute myeloid leukaemia to stem cells. Nature. 2017;547(7661):104‐108. doi:10.1038/nature22993 28658204

[15] van Rhenen A , Feller N , Kelder A , et al. High stem cell frequency in acute myeloid leukemia at diagnosis predicts high minimal residual disease and poor survival. Clin Cancer Res. 2005;11(18):6520‐6527. doi:10.1158/1078-0432.CCR-05-0468 16166428

[16] Thomas D , Majeti R . Biology and relevance of human acute myeloid leukemia stem cells. Blood. 2017;129(12):1577‐1585. doi:10.1182/blood-2016-10-696054 28159741 PMC5364335

[17] Ng SW , Mitchell A , Kennedy JA , et al. A 17‐gene stemness score for rapid determination of risk in acute leukaemia. Nature. 2016;540(7633):433‐437. doi:10.1038/nature20598 27926740

[18] Gentles AJ , Plevritis SK , Majeti R , Alizadeh AA . Association of a leukemic stem cell gene expression signature with clinical outcomes in acute myeloid leukemia. JAMA. 2010;304(24):2706‐2715. doi:10.1001/jama.2010.1862 21177505 PMC4089862

[19] Morita K , Wang F , Jahn K , et al. Clonal evolution of acute myeloid leukemia revealed by high‐throughput single‐cell genomics. Nat Commun. 2020;11(1):5327. doi:10.1038/s41467-020-19119-8 33087716 PMC7577981

[20] Stetson LC , Balasubramanian D , Ribeiro SP , et al. Single cell RNA sequencing of AML initiating cells reveals RNA‐based evolution during disease progression. Leukemia. 2021;35(10):2799‐2812. doi:10.1038/s41375-021-01338-7 34244611 PMC8807029

[21] Dillon LW , Ghannam J , Nosiri C , et al. Personalized single‐cell proteogenomics to distinguish acute myeloid leukemia from non‐malignant clonal hematopoiesis. Blood Cancer Discov. 2021;2(4):319‐325. doi:10.1158/2643-3230.BCD-21-0046 34258102 PMC8265308

[22] Waanders E , Gu Z , Dobson SM , et al. Mutational landscape and patterns of clonal evolution in relapsed pediatric acute lymphoblastic leukemia. Blood Cancer Discov. 2020;1(1):96‐111. doi:10.1158/0008-5472.BCD-19-0041 32793890 PMC7418874

[23] Lapidot T , Sirard C , Vormoor J , et al. A cell initiating human acute myeloid leukaemia after transplantation into SCID mice. Nature. 1994;367(6464):645‐648. doi:10.1038/367645a0 7509044

[24] Bonnet D , Dick JE . Human acute myeloid leukemia is organized as a hierarchy that originates from a primitive hematopoietic cell. Nat Med. 1997;3(7):730‐737. doi:10.1038/nm0797-730 9212098

[25] Shin DY . Human acute myeloid leukemia stem cells: evolution of concept. Blood Res. 2022;57(S1):67‐74. doi:10.5045/br.2022.2021221 35483929 PMC9057671

