# Supplementary material for: Pursuing dynamics of minimal residual leukemic subclones in relapsed and refractory acute myeloid leukemia during conventional therapy
Source: Cancer Med. 2024 Apr 9;13(7):e7182. doi: 10.1002/cam4.7182 (PMC11002636; doi:10.1002/cam4.7182)
Supplement: Supplementary file 1 — Figure S1. [file CAM4-13-e7182-s001.pdf]

Supplementary Figure 1

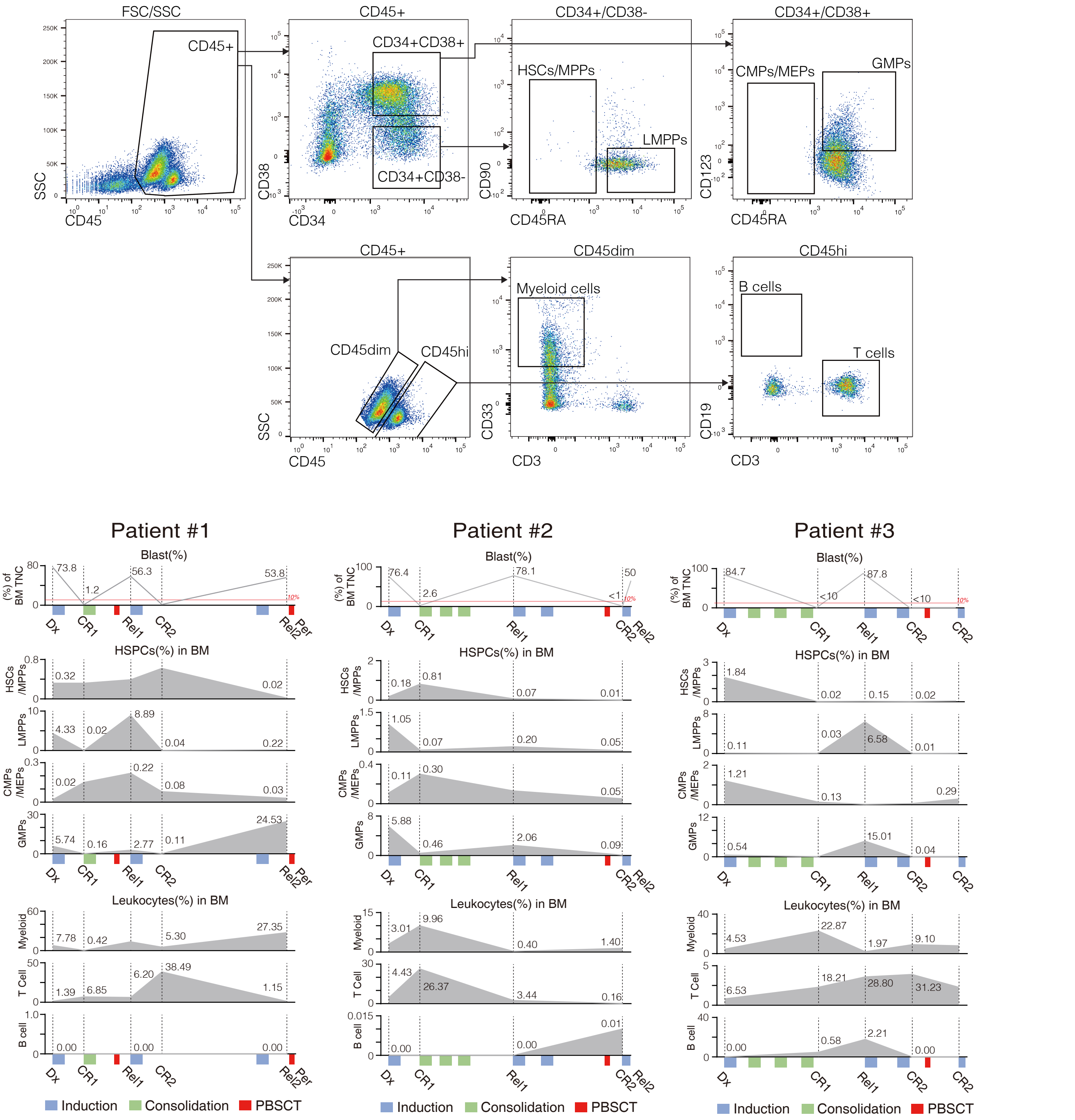

Supplementary Figure 1

Representative flow cytometer plots of the multiparameter immunophenotyping panel. For analysis of HSPC populations, HSC/MPP, LMPP, CMP/MEP, and GMP fractions and mature leukocytes, CD45dim myeloid cells, and CD45 bright (hi, high) B/T cells were analyzed and sorted into seven single fractions. Proportion of AML blasts and 7 cell fractions in patient BM during treatment. Blast counts were assessed by diagnostic examination, and 7 cell fractions were analyzed by FACS. (events/a million total events, parent gated cells). Light blue: induction therapy; green: consolidation therapy; red: allogeneic G-CSF-mobilized peripheral blood stem cell transplantation.
