# Supplementary material for: Pursuing dynamics of minimal residual leukemic subclones in relapsed and refractory acute myeloid leukemia during conventional therapy
Source: Cancer Med. 2024 Apr 9;13(7):e7182. doi: 10.1002/cam4.7182 (PMC11002636; doi:10.1002/cam4.7182)
Supplement: Supplementary file 2 — Figure S2. [file CAM4-13-e7182-s002.pdf]

Supplementary Figure 2

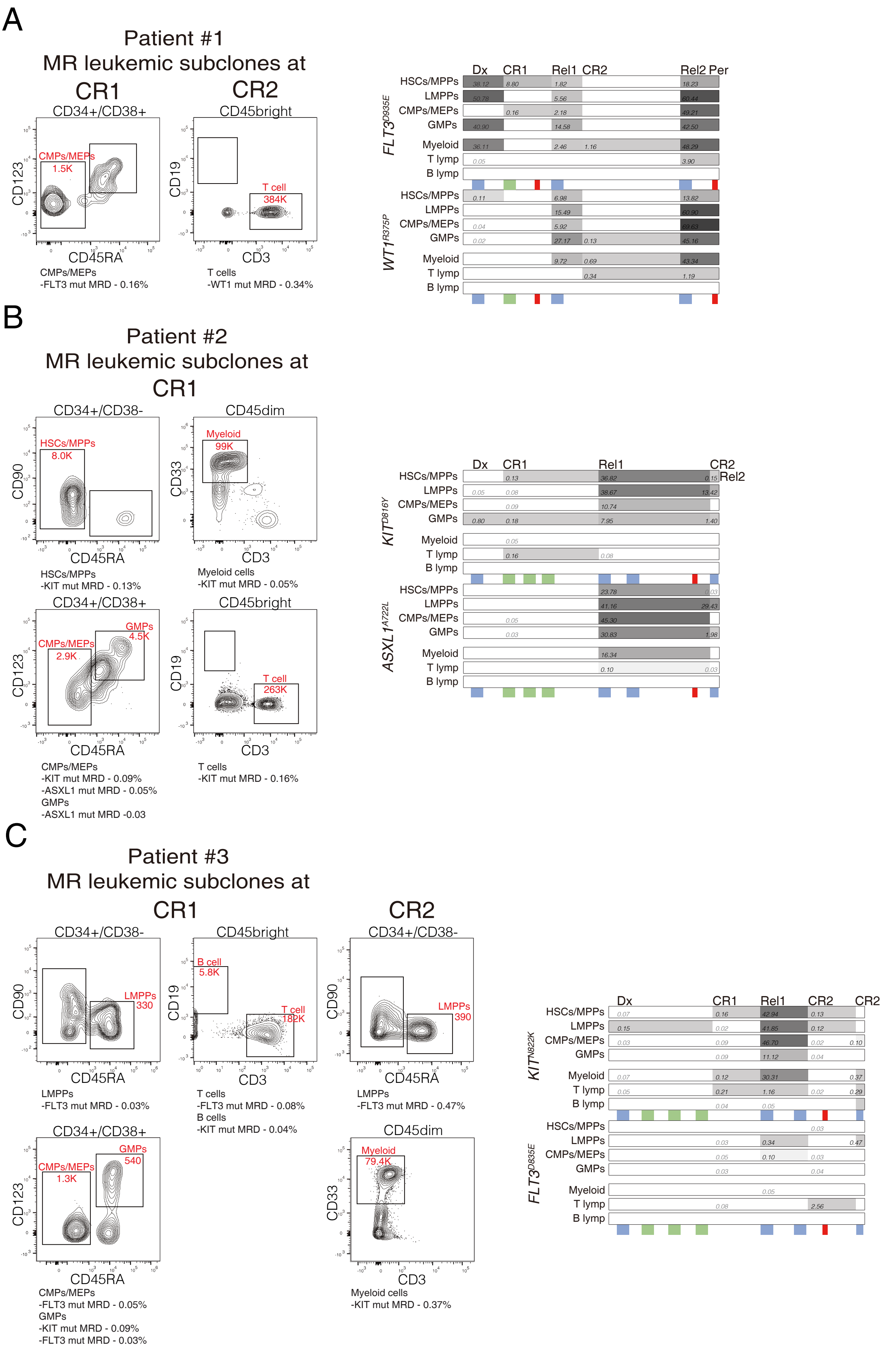

Supplementary Figure 2
FACS plots of cell populations containing treatment-persistent MR leukemic subclones that were newly repopulated at CR of Patients A.#1, B.#2, and C.#3. Numbers in plots indicate events/1 million of total events. Diagrams (right) show the distribution of leukemic subclones according to determined VAF values.
